# Removal, Adsorption, and Cleaning of Pharmaceutical on Polyamide RO and NF Membranes

**DOI:** 10.3390/polym15122745

**Published:** 2023-06-20

**Authors:** Davor Dolar, Iva Ćurić, Danijela Ašperger

**Affiliations:** University of Zagreb Faculty of Chemical Engineering and Technology, Marulićev trg 19, HR-10000 Zagreb, Croatia; icuric@fkit.unizg.hr (I.Ć.); diva@fkit.unizg.hr (D.A.)

**Keywords:** chemical cleaning, adsorption, removal, pharmaceutical, nanofiltration, reverse osmosis

## Abstract

Pharmaceuticals are present in various waters and can be almost completely rejected by membrane separation processes, i.e., nanofiltration (NF) and reverse osmosis (RO). Nevertheless, the adsorption of pharmaceuticals can decrease their rejection, so adsorption can be considered a very important removal mechanism. In order to increase the lifetime of the membranes, the adsorbed pharmaceuticals must be cleaned from the membrane. The used pharmaceutical (albendazole), the most common anthelmintic for threatening worms, has been shown to adsorb to the membrane (solute-membrane adsorption). In this paper, which is a novelty, commercially available cleaning reagents, NaOH/EDTA solution, and methanol (20%, 50%, and ≥99.6%) were used for pharmaceutical cleaning (desorption) of the NF/RO membranes used. The effectiveness of the cleaning was verified by Fourier-transform infrared spectra of the membranes. Of all the chemical cleaning reagents used, pure methanol was the only cleaning reagent that removed albendazole from the membranes.

## 1. Introduction

Pharmaceuticals are found in wastewater, surface water, drinking water, and groundwater worldwide at concentrations ranging from very low (ng/L) (surface water, groundwater) to much higher (mg/L) (hospital and municipal wastewater) [1,2,3,4]. Nanofiltration (NF) and reverse osmosis (RO) have been recognized as promising technologies for the rejection of pharmaceuticals [5,6,7]. Since there are many publications on this topic, Fonseca Couto et al. (2018) [8] and Cevallos-Mendoza et al. (2022) [9] summarized the removal of different contaminants (antibiotics, antidepressants, antihistamines, lipid regulators, non-steroidal anti-inflammatory drugs, pesticides, etc.) using membrane separation processes (NF and RO). The presented reviews showed that RO membranes are the most efficient in removal and show the highest removal (mainly more than 90–95% from synthetic waters). In the case of NF membranes, moderate to high removal of pharmaceuticals was reported [8,9]. The major difference in removal between NF membranes themselves and the RO and NF membranes lies in the removal mechanisms. Bellona et al. (2004) [10] summarized the main removal mechanisms in RO/NF, including size exclusion, Donnan effect (electrostatic exclusion), and hydrophobic interactions. In the case of RO, the main removal mechanism is size exclusion since the molecular weight cut-off (MWCO) of RO membranes is 100 Da, while the molecular weight of most pharmaceuticals is larger, i.e., 150–500 g/mol [8]. Nevertheless, it has been reported that the membrane material may have an effect on the removal of pharmaceuticals with RO membranes. Kimura et al. (2004) [11] showed that polyamide (PA) RO membranes have higher rejection of uncharged pharmaceuticals than cellulose acetate (CA) RO membranes. The main conclusion was that for PA membranes, size exclusion is important, whereas, for CA membranes, polarity determines the rejection.

The first reason for the larger differences in NF membranes is the larger MWCO, which is 100–2000 Da for commercially available membranes [12]. In addition, the large differences in removal with NF membranes have also been explained by two factors [13]: the first is related to the surface properties of the membranes (hydrophilicity, surface charge, and roughness), and the second is related to the characteristics of the compound (molecular weight, electrical charge, and wettability). Charge exclusion is one of the most important mechanisms for organic compounds (especially charged ones) since NF membranes have functional C=O and N–H groups that give them a negative charge [14].

The last mechanism (hydrophobic interactions) is related to the interactions between the pharmaceutical and the polymer matrix of the membrane, i.e., adsorption. Adsorption depends on the chemical-physical properties of the pharmaceutical (e.g., water solubility, hydrophobicity, charge, etc.), the solution (e.g., pH), and the membrane (e.g., hydrophobicity/hydrophilicity, roughness, etc.). In general, hydrophilic compounds (Log *K*_O/W_ below 2.5) are thought to be effectively rejected by the size exclusion mechanism because these compounds do not adsorb to the membrane [15]. On the other hand, hydrophobic interactions between the aromatic rings and the hydrocarbon chain present in the organic compounds and the surface active layer of RO membranes have also been found to impact the rejection due to adsorption on the membrane surface [13].

Albendazole (chemical name methyl [5-(propylthio)-1*H*-benzoimidazole-2-yl] carbamate) is one of the most important anthelmintics. It is used against various types of parasites, i.e., it is used to treat certain infections caused by worms such as the pork tapeworm and the dog tapeworm and also in humans [16,17,18]. Albendazole and its active metabolites are very easily released into the environment as they are excreted in urine and/or feces [19]. Higher consumption results in higher concentrations in municipal wastewater and, consequently, higher inputs of albendazole to receiving waters. To the authors’ knowledge, there is no work addressing the removal of albendazole from different water matrices. Our previous study [20] showed complete removal (>99.9%) of albendazole by RO (LFC-1 and XLE) and tight NF90 membranes. However, the removal was much lower (36.1–49.4%) for loose NF membranes (NF270, NF, and DK), which may be attributed to the more open structure of these membranes. 

The surface and pores of membranes provide space for the adsorption of compounds, especially hydrophobic compounds [15,21,22,23,24,25]. In particular, the feed concentration [11,26], retention [11,27,28], and flux [29,30] decrease. On the contrary, the concentration of monitored compounds in the permeate stream increases with time [11,27,31].

To preserve the properties of the membranes, it is important to clean them with chemical cleaning reagents. Often their composition is unknown. The most important reagents are alkalis, acids, metal-chelating agents, surfactants, and enzymes [32]. Acidic and alkaline cleaning agents are used for precipitated inorganic substances (scaling) and organic substances, respectively [33]. 

The effects of chemical cleaning on permeability, pore size, surface charge, surface morphology, and hydrophobicity of NF/RO membranes have been studied previously [32,34,35,36,37,38,39,40,41]. The effects of chemical cleaning on pharmaceutical removal have been studied in detail [35,37,38,39,40,41]. 

In our previous work [42], five pharmaceuticals (albendazole, procaine·HCl, hydrocortisone, trimethoprim, and sulfaguanidine) were used to determine adsorption on the membrane. Adsorption was determined using rejection and Fourier transformation infrared (FTIR) spectra. According to the log *K*_O/W_, only albendazole (3.07) was hydrophobic, while all other compounds were hydrophilic. The main conclusion of this study was that adsorption of the hydrophobic compound (albendazole) was observed on both RO and NF membranes. During albendazole treatment, the rejection decreased with time and the FTIR spectra showed new peaks. The adsorption phenomenon was less pronounced on the RO membrane due to the smaller pore size. For hydrophilic compounds, no adsorption occurred on the RO membranes. Nevertheless, H-bonding and π–π interactions were detected between procaine and the NF270 membrane, showing that hydrophilic compounds can also be adsorbed on the polymeric matrix of the membrane. Since hydrophobic compounds showed very intense adsorption, we decided to use them in the next studies aimed at removing the adsorbed pharmaceuticals from the membrane to restore the membrane properties.

To the authors’ knowledge, there is no study dealing with NF/RO membranes cleaning after pharmaceutical adsorption. Therefore, two main objectives were set in this experimental study: (i) to determine the removal efficiency and mechanism of albendazole on NF/RO membranes using concentration, rejection (*R*), and FTIR spectra and (ii) to desorb albendazole from the studied membranes by chemical cleaning using a commercial chemical cleaning reagent, sodium hydroxide (NaOH) and ethylenediaminetetraacetic acid (EDTA) solution, and methanol (20, 50, and ≥99.6%).

## 2. Materials and Methods

### 2.1. Membranes and Chemicals

The reverse osmosis membranes used were BW30 and XLE from Dow-Filmtec (Midland, TX, USA) and UTC-70HA from Toray (Tokyo, Japan) with a MWCO of 100 Da. The nanofiltration membranes used were NF, NF90, and NF270 from Dow-Filmtec (Midland, TX, USA) with a MWCO of 150–300 Da. The manufacturer stated that the pH range for operation is 2 to 11. However, for cleaning, the pH range can be extended to pH 1 and 12 (13, depending on the membrane), keeping in mind that this is a short-term cleaning, i.e., 30 min. For all membranes used, the maximum operating temperature must not exceed 45 °C but must not exceed 35 °C for pH values above 10. The contact angles of XLE, NF, NF270, and NF90 membranes were 51.3°, 51.7°, 69.0°, and 66.2°, respectively, indicating intermediate contact angles.

Albendazole (analytical grade, >99%, Veterina (Kalinovica, Croatia)) is a hydrophobic pharmaceutical and a 15 mg/L stock solution was prepared in ultrapure water. Table 1 shows the main characteristics of albendazole.

### 2.2. Infrared Spectrometer with Fourier Transformation and Goniometer

The FTIR spectra of the pristine and the fouled membranes were measured using a Bruker Vertex 70 FTIR spectrometer (Ettlingen, Germany) equipped with a Platinum ATR single reflection diamond (*n* = 2.4) crystal-based module in the mid infrared range (400–4000 cm^−1^). The infrared spectra of the membranes were recorded with a resolution of 4 cm^−1^ and 32 scans.

The contact angle was determined by the sessile drop method using a DataPhysics OCA 20 goniometer (Filderstadt, Germany). A drop of MilliQ water was manually formed using a micrometer doser, and the drop image was captured using the camera built into the instrument. The volume of the drop was 2 μL at room temperature. To minimize experimental error contact angles were measured at five random locations for each sample and the average value was reported (*N* = 5).

### 2.3. Analytical Methods

Albendazole concentrations in feed and permeate were analyzed using Varian ProStar 500 (Walnut Creek, CA, USA) high performance liquid chromatography (HPLC). This system consisted of a ProStar 410 autosampler, a ProStar 230 tertiary pump system, a ProStar 330 diode array detector (DAD), and a thermostatically controlled column compartment. HPLC separations were performed on an InertSustain^TM^ C18 column (250 mm × 4.6 mm, 5 µm) (GL Sciences Inc., Fukushima, Japan). In this analysis, 0.01% formic acid in MilliQ water was used as eluent A and 0.01% formic acid in acetonitrile was used as eluent B in gradient elution mode. After gradient elution, the column was equilibrated for 5 min before another injection. The flow rate was 0.5 mL/min. An injection volume of 30 μL was used for all analyses. Albendazole was measured at 210 nm (*t*_R_ = 22.720 min) on a DAD. The column temperature was 30 °C. The method detection limit (MDL) was determined by analyzing spiked MilliQ water samples as concentration and a S/N ratio of 3. For the pharmaceutical analyzed, the MDL was 0.006 mg/L.

### 2.4. Membrane Cleaning Reagents

Nalco PermaClean99 (Nalco PC99, Oegstgeest, The Netherlands), a commercial cleaning reagent, NaOH/EDTA mixture, and methanol were used to remove adsorbed albendazole from fouled membranes. Nalco PC99 in liquid form (1.5% *w*/*w*) gave a transparent solution in demineralized water with a pH of 12.23. The main component of Nalco PC99 is potassium hydroxide (5% *w*/*w*). The second cleaning solution was prepared with NaOH (0.1% *w*/*w*) and EDTA (0.1% *w*/*w*) in demineralized water. Cleaning was also performed with methanol (20%, 50% and ≥99.8%). 

### 2.5. RO/NF Laboratory System

This study was performed out in a laboratory apparatus described in detail by Dolar et al. 2011. [44]. The apparatus consists of six cells connected in parallel. All experiments were performed under the following basic conditions: working pressure, flow rate, and membrane surface area of 10 bar, 750 mL/min, and 11.0 cm^2^, respectively.

To clean the pristine membranes from the conserving agents, they were washed without pressure with about 7 L of demineralized water. The membranes were pressurized at 15 bar for 2 h to stabilize membrane permeate flux. Albendazole solutions (pH = 5.8–6.2) treatment lasted 4 h in batch circulation mode (permeate and retentate streams circulated back to the feed stream). The feed stream was sampled hourly, while permeate samples were collected twice, i.e., at 0 h and 4 h. Membranes were cleaned with Nalco PC99, NaOH/EDTA, and methanol (20% and 50%) at elevated temperature (*T* = 31.5 ± 2.2 °C) and pH = 12.08 ± 2.19. The cleaning procedure was as follows: (1) circulating the cleaning solution at working pressure and elevated temperature for 30 min, (2) soaking the membranes in the cleaning solution for 30 min, and (3) washing the membranes with a large volume (at least 30 L) of demineralized water. All membrane samples (pristine and cleaned) were dried at 35 °C for about 15 h to remove residual water. All these membrane samples were analyzed by FTIR within a maximum of 5 days. Cleaning with pure methanol was performed by soaking the adsorbed membranes in methanol for 1 h. After soaking, the membranes were washed manually with demineralized water.

## 3. Results and Discussion

### 3.1. Rejection Factor and Removal Mechanism of Albendazole on Used Membranes

The rejection factors obtained at the beginning (0 h) of albendazole treatment with the membranes studied are shown in Table 2. The rejection of albendazole was very similar for all membranes used (approximately 78%), with the exception of NF270 (65.4%), and is in agreement with previous results published in Dolar et al., 2017. [42]. Size exclusion is one of the most important removal mechanisms in the rejection of pharmaceuticals from water and can be explained in two ways. This mechanism can be assumed when the MWCO of the membranes and the molecular weight of the compound are compared. Also, the pore size of the membrane could be compared with the length, width, and/or height of the molecule. Depending on the molecular weight of albendazole and MWCO of the membranes, the rejection must be higher, especially for RO membranes. The MWCO of all membranes studied was lower than the molecular weight of albendazole (265.33 g/mol), except for NF and NF270 (the MWCO of RO and tight NF membranes is 100 Da and of loose NF membranes is 150–300 Da).

Furthermore, depending on the compound and membrane properties, electrostatic repulsion can be assumed to be an additional removal mechanism for size exclusion. This is due to the charge of the membrane and compounds under working conditions (pH). It is known that the charge of RO/NF membranes becomes more negative with increasing pH. Depending on the source, some of the membranes used in this study had a zeta potential between −2.93 and −8.92 mV [42] and between −16 and −27 mV [45]. Both removal mechanisms result in almost complete removal with RO and NF90 membranes, as shown in our previous study [20]. However, the rejection factors in this study were lower due to the 15-fold higher concentration, the size of the compound, and a third removal mechanism (hydrophobic interactions/adsorption). According to the dipole moment of albendazole (which indicates the direction of the compound to the membrane) [42], width (0.482 nm), and height (0.279 nm) were the most important parameters of albendazole to be compared with the pore sizes of the membranes used. Since the pore size of RO membranes is in the range of ~0.3–0.35 nm and for NF membranes in the range of 0.5–2 nm [13], the lower rejection of albendazole is visible. According to this comparison, albendazole is able to enter the pores of the membranes and penetrate through the membrane. This is confirmed by the increase in albendazole concentration in the permeate stream (Table 2). However, adsorption on the polymer matrix of the membrane (surface and pores) is probably the most important reason for the lower rejection. Albendazole is hydrophobic (Log *K*_O/W_ = 3.07), so it can be easily adsorbed. 

The adsorption of albendazole was determined using *R*, concentrations (permeate and feed), and FTIR spectra. Table 2 shows the *R* value and concentrations of albendazole in permeate at 0 and 4 h of treatment, while Table 3 shows the concentrations of albendazole in feed throughout the treatment. Due to the hydrophobic interactions [46,47] and the direction of the dipole moment (indicating the possibility of penetration of the substance into the pores of the membranes), the rejection of albendazole was similar for all membranes used. The continuous decrease in albendazole concentration in the feed is due to the short duration of the experiment (4 h) and indicates that the steady state was not reached. In our previous study [48], the results confirmed adsorption, and for some compounds (hydrocortisone and dexamethasone) the steady state in feed concentration was reached after 12 h. In the present study, the aim was not to reach steady state but to foul the membrane, i.e., to adsorb albendazole to the membrane and clean it.

Since the log *K*_O/W_ of albendazole is 3.07 (hydrophobic organic compound), it tends to interact with the membrane polymer, i.e., adsorb on the surface and in the pores [21,27,31]. Adsorption significantly decreased the rejection efficiency of albendazole in all membranes studied [26,27,28], which was related to the increase in albendazole concentration in the permeate [11,27,31]. After a 4 h treatment, the decrease in rejection was highest for loose nanofiltration membranes (19–29%), while it ranged from 13% to 16% for RO and tight NF90 membranes. It can be concluded that the rejections between these two groups of membranes were almost equal. Due to the dipole moment and adsorption, albendazole passed through the membranes, indicating an increase in permeate concentration (Table 2).

### 3.2. Chemical Cleaning of Adsorbed Albendazole on RO/NF Membranes

**Figure 1:**
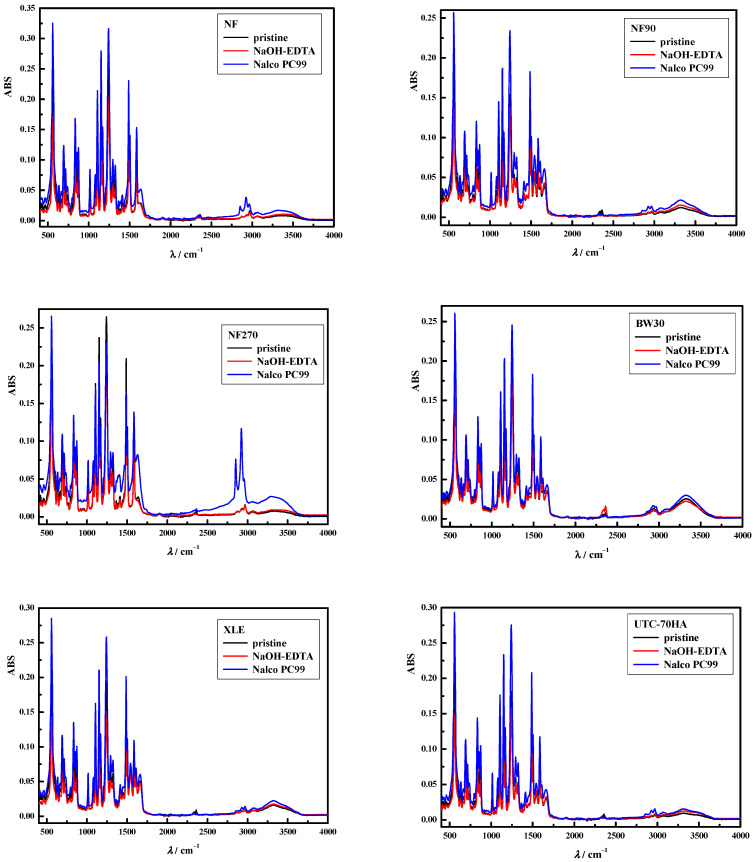
Effect of cleaning agents (NaOH/EDTA mixture and Nalco PC99) on the membrane structure.

First, the effect of used cleaning agents on the membrane structure was investigated and is shown in Figure 1. For most membranes, the FTIR spectra showed no discernible changes in the peaks after the membranes were exposed to chemical cleaning solutions. By changes, the authors mean that no new peaks were formed or that the existing peaks were removed. Fujioka et al. (2014) [35] showed no changes in the FTIR spectra of the membranes after chemical cleaning with sodium hydroxide, Floclean MC11, PermaClean PC98, etc. The presented results confirmed that no hydrolysis of the polyamide skin of the membranes occurred.

In our study, only the NF and NF270 membranes showed minor changes at higher wavelengths (>2800 cm^−1^). In these pristine membranes, small peaks are present between 2850 cm^−1^ and 3000 cm^−1^ and at 3300 cm^−1^. After treatment with Nalco PC99, the peaks between 2850 and 3000 cm^−1^ were more pronounced and are attributed to greater C-H stretching. The second change was the broad peak at 3300 cm^−1^ that was pronounced for the NF270 membrane. This indicates that abundant –OH and –NH groups were present on the surface of the NF270 membrane after Nalco PC99. 

**Figure 2:**
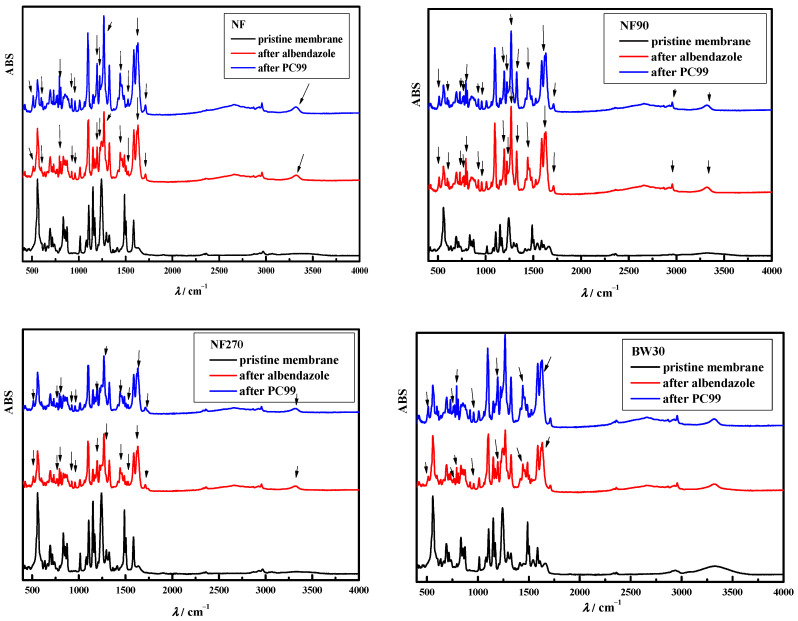

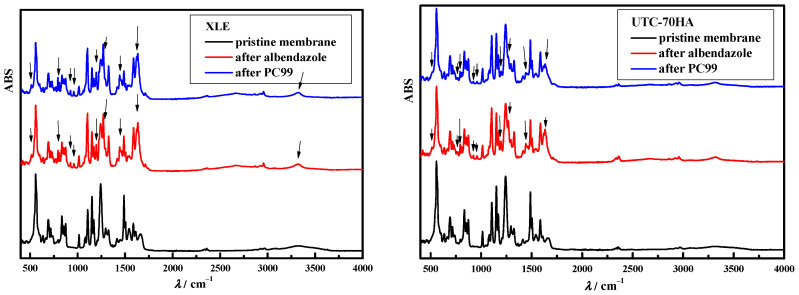
FTIR spectra of pristine membranes, membranes after albendazole treatment, and after cleaning with Nalco PC99 (arrows represent changes, i.e., new peaks, on membrane FTIR spectra compared to pristine membrane).

**Figure 3:**
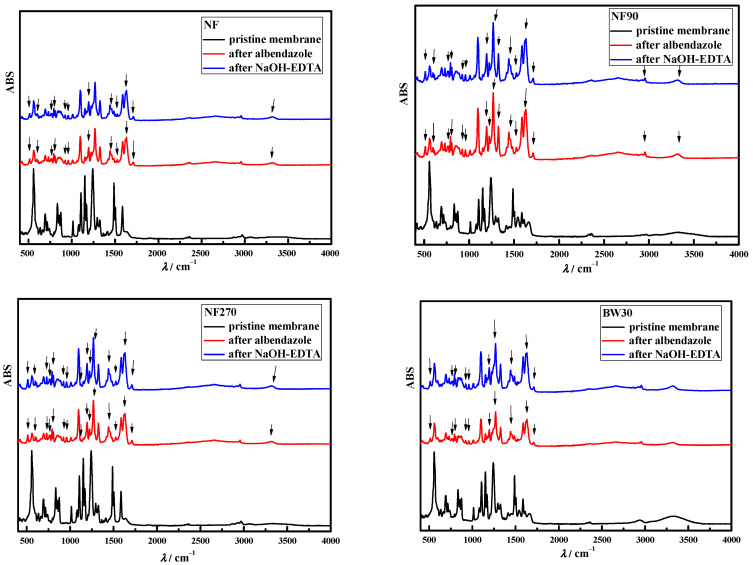

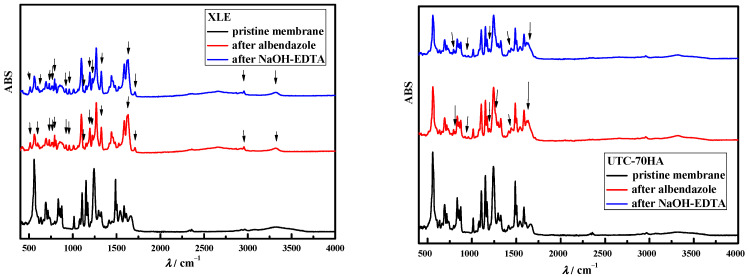
FTIR spectra of pristine membranes, membranes after treatment with albendazole, and after cleaning with NaOH/EDTA mixture (arrows represent changes, i.e., new peaks, on membrane FTIR spectra compared to pristine membrane).

The changes in FTIR spectra during treatment with albendazole solution and after cleaning are shown in Figure 2. (Nalco PC99) and Figure 3. (NaOH/EDTA). The black line represents the pristine membranes, the red line represents the membranes after treatment with albendazole, and the blue line represents the membranes after cleaning (Nalco PC99 in Figure 2 and NaOH/EDTA solution in Figure 3). The difference between the pristine membranes and the membranes after treatment with albendazole strongly indicates adsorption on their structure. New peaks (with arrows pointing up), i.e., bonds, were present.

The interactions between albendazole and the membrane are shown in Figure 2 and Figure 3 (red line in the FTIR spectra). For all membranes studied, direct H-bonding between the H of the OH group and the nitrogen of the heterocyclic ring (3320 cm^−1^) was observed and was still present after cleaning. For the BW30 and XLE membranes, the entire baseline shifted upward (range 2150–2820 cm^−1^), indicating that cleaning did not efficiently desorb albendazole bound on the membrane surface (this range shows the binding of albendazole to the membrane surface due to its physico-chemical characteristics [42]). The orientation of the dipole moment of albendazole allows the 2-methylcarbamoyl substituent to enter the membrane structure while the ethyl group remains at the end of the molecule (aliphatic part). At 771 and 792 cm^−1^, CH_2_ bending was observed in the aliphatic part of albendazole before and after cleaning. Moreover, according to Figure 2, new bonds of the carbonyl group of albendazole (1620 and 1632 cm^−1^), stretching of the double bond C=C in an aromatic ring located in the polyamide top layer (1400–1525 cm^−1^), bonds C(sp^2^)–O–C(sp^3^) (1269 cm^−1^), bending bonds C–O–C (1194 cm^−1^), and string bending of the methyl group (–CH_3_) of albendazole (800–1000 cm^−1^) were detected. 

The same results, i.e., new peaks, were obtained for the studied NF membranes due to their similar structure. From the red lines in Figure 2 and Figure 3, it is clear that most of the adsorption occurred in the polysulfone and polyamide layers, as the peaks in the range between 800 and 1800 cm^−1^ are assigned to the polysulfone and polyamide layers, while the fingerprint region between 1500 and 1800 cm^−1^ includes carbonyl groups and amide bands in the polyamide group [49].

As can be seen in Figure 2 and Figure 3 (blue lines), not all peaks formed in the polymer matrix of the membranes after albendazole treatment were removed. Both the commercial Nalco PC99 and the NaOH/EDTA mixture were unable to remove the adsorbed pharmaceutical from the membrane. The penetration of albendazole into the polymer matrix, combined with its poor water solubility and hydrophobic nature, led to the formation of new bonds, i.e., interactions. All the cleaning agents used were water-based and probably not effective in desorbing albendazole from the polymer matrix. In addition, the pH of all the cleaning agents used was high (>12), which could affect the solubility coefficient of albendazole. Torrado et al. (1996) [50] showed that the solubility coefficient of albendazole increased with decreasing pH (at pH 1.2 and 6.0, it was 0.376 mg/mL and 0.016 mg/mL, respectively). Thus, the alkaline pH of the cleaning was twice the maximum value used by Torrado et al. (1996) [50], although it can be assumed that the solubility of albendazole in the cleaning agents used here was even lower.

**Figure 4:**
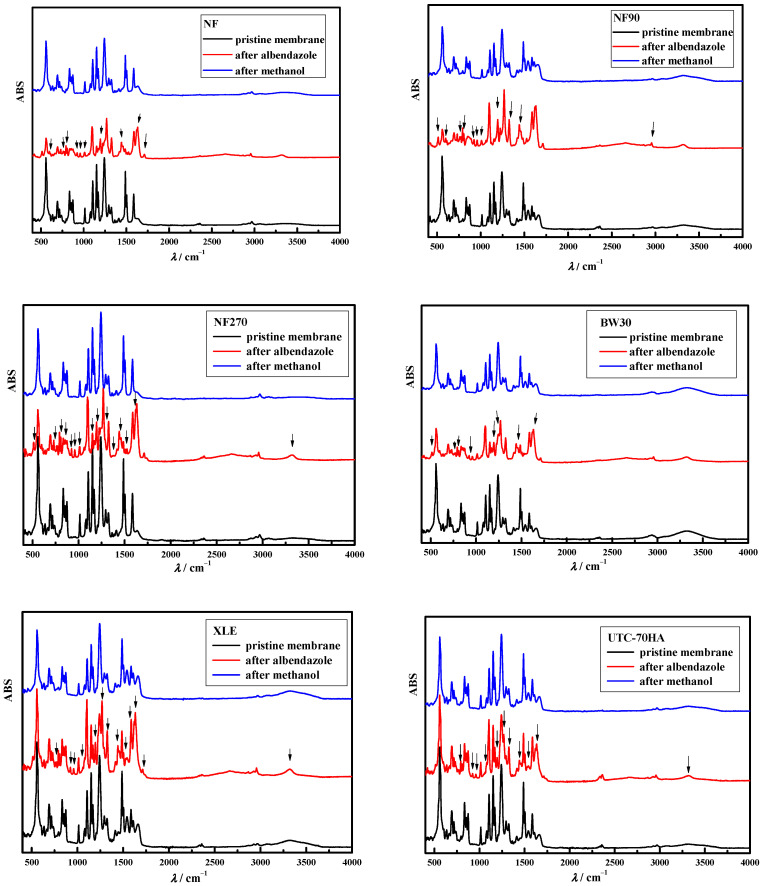
FTIR spectra of pristine membranes, membranes after treatment of albendazole, and after cleaning with pure methanol (arrows represent changes, i.e., new peaks, on membrane FTIR spectra compared to pristine membrane).

Adsorption phenomena affect the overall removal of pharmaceuticals (especially hydrophobic ones) with NF and RO membranes. Therefore, the second goal of this study was to find a suitable cleaning solution for the desorption of pharmaceuticals from the membrane. Most pharmaceuticals are highly soluble in methanol. Therefore, methanol concentrations of 20%, 50%, and ≥99.8% were used. The concentrations of 20% and 50% were not effective in desorbing albendazole from the membranes used because newly formed peaks were still present in the FTIR spectra of the membranes (results not shown here). The results were similar to those obtained with the Nalco PC99 and NaOH/EDTA mixture cleaning agents. The results for the pristine membranes, after treatment with albendazole solution and after soaking in pure methanol, are shown in Figure 4. 

As before, the red lines in Figure 4 show new peaks confirming adsorption in the polymer matrix of the membranes studied. However, the blue lines (after soaking in methanol) show that the new peaks were removed, i.e., the albendazole was desorbed from the NF/RO membranes studied.

The results presented in this study show that pure methanol is the only cleaning agent that effectively desorbs albendazole and that the solubility of the pharmaceutical has an effect on its desorption from the polymer membrane matrix. Nevertheless, pure methanol is not a very good cleaning agent because it is hazardous to humans and the environment. When methanol is released into the environment, it rapidly breaks down into other compounds, is completely miscible with water, and serves as food for a number of bacteria. Therefore, it is very important to spend extra energy to find a suitable and effective cleaning agent for the desorption of various pharmaceutical substances, especially hydrophobic substances from the polymer matrix of the membrane. 

## 4. Conclusions

Albendazole desorption was confirmed by concentrations that decreased in the feed and increased in the permeate. In addition, the rejection efficiency decreased with treatment time and new peaks were found in the FTIR spectra of the membranes. FTIR spectra were used to confirm the effectiveness of chemical cleaning of the membranes. After cleaning with chemical reagents (commercial, NaOH/EDTA solution, and 20% and 50% methanol), the formed bonds were still present. Only pure methanol completely desorbed albendazole from the polymer matrix of the membrane.

## Figures and Tables

**Table 1:** Physico-chemical characteristics of albendazole.

		Molecular Structure	Direction of Dipole Moment
CAS number	54965-21-8	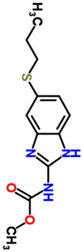	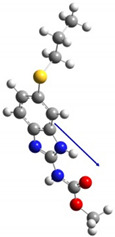
Molecular weight, M_w_ (g/mol)	265.33		
Water solubility (mg/L) ^1^	46.39		
width (nm) ^2^	0.482		
height (nm) ^2^	0.279		
length (nm) ^2^	1.632		
log *K*_O/W_	3.07		
log *D* (pH = 7.4) ^3^	3.06		
p*K*_a_^4^	6.90		
Dipole moment, *μ* (D) ^5^	4.33		
Charge at pH 7	negative		

Characteristics obtained from EPI SUITE^TM^ v4.10 (otherwise noted); ^1^ estimated values; ^2^ calculated with HyperChem 8.0; ^3^ obtained from www.chemspider.com (accessed on 8 March 2021); ^4^ obtained from the Syracuse Corporation (SRC) PhysProp database (http://esc.syrres.com/fatepointer/search.asp, accessed on 8 March 2021); ^5^ dipole moment calculated by Gaussian [43].

**Table 2:** Rejection and concentrations of albendazole in permeate (*γ*_p_) at the beginning (0 h) and the end (4 h) of the treatment.

MWCO *t*/h	UTC-70HA 100	XLE 100	BW30 100	NF90 100	NF 150–300	NF270 150–300
	*R*/%					
0	78.1	78.6	78.5	79.1	73.8	65.4
4	61.2	63.7	62.8	62.4	44.8	43.8
	*γ*_p_/mg/L					
0	3.39	3.32	3.33	3.24	4.06	3.24
4	3.91	3.66	3.75	3.79	5.56	3.79

**Table 3:** Concentrations of albendazole in the feed stream (***γ*_F_**) during whole treatment.

*t*/h	*γ*_F_/mg/L
0	15.50
1	12.76
2	12.41
3	11.96
4	10.08

## Data Availability

Not applicable.
